# Health, education and well-being for children with deafblindness: a secondary analysis of 36 Multiple Indicator Cluster Surveys

**DOI:** 10.1136/archdischild-2025-328675

**Published:** 2025-04-17

**Authors:** Sara Rotenberg, Calum Davey, Lena Morgon Banks

**Affiliations:** 1 Faculty of Epidemiology and Population Health, London School of Hygiene and Tropical Medicine, London, UK

**Keywords:** Healthcare Disparities, Adolescent Health, Child Health, Global Health

## Abstract

**Objective:**

To examine the health, education and social inequities experienced by children with deafblindness in low- and middle-income countries.

**Design:**

Secondary analysis of 36 Multiple Indicator Cluster Surveys (2017–2020), using age-adjusted modified Poisson models to compare outcomes between children with and without deafblindness.

**Setting:**

36 low- and middle-income countries.

**Patients:**

446 233 children aged 2–17, including 232 children with deafblindness.

**Main outcome measures:**

Education (primary school attendance rate, secondary school attendance rate, early childhood education and the Early Childhood Development Index), health (stunting, wasting, health insurance, diarrhoeal disease and acute respiratory infection) and well-being (inadequate supervision, violent discipline, living arrangements, birth registration and poverty status) were measured.

**Results:**

Children with deafblindness faced inequities in health and education indicators compared with children with other disabilities and children without disabilities. Children with deafblindness had consistently lower school attendance rates across school ages (primary: adjusted Prevalence Ratio (aPR) 0.30 (0.18 to 0.50); secondary: aPR 0.42 (0.20 to 0.87)), had worse Early Childhood Development Indices (aPR 0.35 (0.22 to 0.55)) and had 2–3 times higher prevalence of nutritional disorders (stunting: aPR 1.24 (1.03 to 1.50); wasting: aPR 2.79 (1.99 to 3.92)). However, there were non-significant differences in well-being indicators, such as poverty, inadequate supervision, birth registration and living situation. Children with deafblindness were also less likely to experience violent discipline.

**Conclusion:**

Children with deafblindness constitute a heterogeneous group of children with disabilities. They face persistent barriers in accessing education and have poorer health, which must be addressed through building more disability-inclusive health and education systems.

**WHAT IS ALREADY KNOWN ON THIS TOPIC**

- Two recent global reports by the World Federation of the Deafblind have highlighted significant inequities in education, health and well-being of people with deafblindness globally.
- There are persistent barriers to equal and full participation for people with deafblindness, as well as a lack of data and research on this marginalised population, particularly children with deafblindness and those living in low- and middle-income countries.

**WHAT THIS STUDY ADDS**

- This study adds new insights into the education, health and well-being of children with deafblindness across 36 low- and middle-income countries. It provides novel evidence that children with deafblindness are significantly less likely to be in school or on track for development, as well as more likely to experience nutritional disorders or diarrhoeal disease.

**HOW THIS STUDY MIGHT AFFECT RESEARCH, PRACTICE, OR POLICY**

- These results highlight that children with deafblindness face exceptional barriers in education and worse health outcomes. This suggests that they are not adequately considered in policy measures, and further research should be done to examine possible interventions to improve the education and health status of children with deafblindness.

## Introduction

 Deafblindness is defined as ‘a distinct disability arising from dual sensory (hearing and vision) impairment of severity that makes it hard for the impaired senses to compensate for each other’.^1^ The term ‘deafblindness’ encompasses various levels of impairment, ranging from complete loss of both senses to varying degrees of residual hearing and/or vision.^2^ Deafblindness can be congenital or acquired, and it is estimated that approximately 0.2%–2% of the world’s population are deafblind.^1^ The prevalence of deafblindness increases rapidly with age.^3^ Still, in an analysis of data from nationally representative, population-based surveys from 36 low- and middle-income countries, it is estimated that over 1.8 million children (0.9% of the total child population) are deafblind (including mild, moderate or severe deafblindness).^1^

Deafblindness is often not recognised as a distinct disability.^1^ However, children with deafblindness experience unique barriers to participation in society, particularly in education, healthcare and social inclusion.^1^ For example, people with deafblindness often have specific support requirements, including interpreter guides or tactile or tadoma (based on jaw/facial movements) communication.^1^ However, these services are in short supply or are not adequately covered under existing legislation given the lack of recognition of deafblindness as a distinct disability in many settings: in a survey of stakeholders from 50 countries, only 30% of countries reported that interpreter guides were available and only 20% paid by government.^1^ Coverage was particularly poor in low- and middle-income countries, with only 6%–15% of countries surveyed reporting that interpreter guides were available. Accessing services even when they are available can be complicated by costs and poor information. For example, data from the USA has shown there are very low referral rates to specialised services for children with deafblindness before the age of three, despite the importance of these early childhood development services.^4^

Lack of access to required goods and services, combined with other barriers such as stigma, can have profound impacts on people with deafblindness. For example, children with deafblindness are often excluded from mainstream educational institutions, as few educational institutions have the necessary supports (ie, assistive technology, deafblind interpretation, trained teachers and other support staff, etc)^5^ to adequately include these children.^6 7^ Mainstream early childhood education and high school graduation rates for children with deafblindness in the USA have increased substantially in the past decades, though overall attendance remains low.^8^ Furthermore, some studies among adults with deafblindness in high-income countries have reported worse mental and physical health, lower trust and feeling socially isolated compared with people without disabilities.^9 10^

Similarly, people with deafblindness have reported barriers to accessing healthcare, particularly with regard to the inaccessibility of physical environments, costs of accessing care, accessing information about health and healthcare in an accessible format and communicating with healthcare providers.^11^,^13^ These barriers may be further exacerbated by additional impairments, as a study in Canada showed 86% of children with deafblindness have additional disabilities.^14^ These difficulties in accessing healthcare likely contribute to worse health status and higher mortality among people with deafblindness.^15 16^ For example, US data showed that older adults with deafblindness were three times more likely to have self-rated poor health^17^ and higher mortality.^15^

Overall, there are major gaps in evidence on the health, education and well-being of children with deafblindness in low- and middle-income countries. The majority of academic literature comes from adults, high-income settings and qualitative work.^18 19^ Several reasons may explain this lack of evidence, including lack of measurement of deafblindness, inadequate power to disaggregate surveys, limited recognition of deafblindness as a distinct disability rather than ‘multiple disabilities’ and limited researchers and engagement with the deafblind community.^18^ Therefore, this paper used data from 36 internationally comparable UNICEF-supported Multiple Indicator Cluster Surveys (MICS)^20^ to examine common health, education and well-being indicators for children with deafblindness compared with children with other disabilities and children without disabilities.

## Methods

### Data

The MICS are nationally representative household surveys that measure the well-being of children and women in low- and middle-income countries.^20^ We included the sixth round of the available MICS conducted between 2017 and 2020, specifically those surveys with Washington Group questions to assess functioning among children 2–17. In total, we included data from 36 countries across several UNICEF regions, including Central and South Asia (n=4), Eastern and Southern Asia (n=1), Latin America and the Caribbean (n=7), North Africa and West Asia (n=5), North America and Europe (n=3), Oceania (n=3) and Sub-Saharan Africa (n=13). The sample included 446 233 children, including 232 children with deafblindness and 50 762 children with other disabilities.

### Measurement of disability and deafblindness

Surveys in the sixth round of the MICS include data on child functioning for children 2–17. There are two UNICEF/Washington Group modules to assess child functioning: one for children aged 2–4 and another for children aged 5–17. For both, the child’s caregiver is asked about the level of difficulty that their child faces in doing daily activities, including seeing, hearing, walking, controlling behaviour, learning and communication compared with other children of a similar age. For children aged 2–4, caregivers are also asked about dexterity and playing, while caregivers of children aged 5–17 are asked about remembering, concentrating, self-care, making friends, accepting change, anxiety and depression. For most questions, there are four response options: no difficulty, some difficulty, a lot of difficulty and cannot do.^21^,^23^

Deafblindness is defined as ‘a combined vision and hearing impairment of such severity that it is hard for the impaired senses to compensate for each other’.^24^ Children were classified as deafblind if their caregiver reported they had at least a lot of difficulty in both the hearing and vision functional domains, in line with the First Global Report on Deafblindness.^1^ Children with deafblindness may have also had other functional difficulties but were recoded as deafblind.

Children were classified as having another disability (other than deafblindness) if they answered ‘a lot of difficulty’ or ‘cannot do at all’ to any of the functional domains except both vision and hearing. Children without disabilities reported no or only some difficulty across the functional domains. Children with missing data for one or more domains who had otherwise not responded that they had difficulty in the other functional domains were excluded from the analysis to reduce non-response bias, as their disability and deafblind status could not be determined.

### Outcomes and covariates

The aim of this paper was to understand the differences in education, health and well-being outcomes for children aged 2–17 with deafblindness. Therefore, our outcomes focused on the available education, health and well-being indicators in the MICS. Outcomes are defined in table 1.

**Table 1:** Indicators used to explore the situation of children with deafblindness

Outcomes	Description
Education	
Primary school attendance rate	Primary school age child (based on country standard) is attending or has completed primary school.
Secondary school attendance rate	Secondary school age child is attending or has completed secondary school.
Early childhood education attendance	Child (3–4 years) attends an early childhood education programme
Early Childhood Development Index	Child (3–4 years) is developmentally on track in 3+ of the following four domains of literacy-numeracy, physical functioning, social-emotional development and learning.
Health	
Stunted	Child (2–4 years) is more than two SD below WHO z-scores for height-for-age.
Wasted	Child (2–4 years) is more than two SD below WHO z-scores for weight-for-height.
Has health insurance	Child has health insurance (any type).
Diarrhoea	Child (2–4 years) experienced diarrhoea in the last 2 weeks.
Acute respiratory infection	Child (2–4 years) had an acute respiratory infection in the last 2 weeks.
Well-being	
Inadequate supervision	Child (2–4 years) has been left alone or under supervision of a child younger than 10 for more than 1 hour at least once in the last week
Violent discipline	Child (2–14 years) experienced physical punishment and/or psychological aggression by caregivers in the last 1 month (caregiver reported)
Lives with both biological parents	Child is living in a household where both his/her biological parents also live
Birth is registered	Child (2–4 years) has a birth certificate or caregiver reports their child is officially registered with a government authority
Lives in poverty	Child lives in a household that is in the bottom 40% of wealth quintiles.

### Statistical analysis

We disaggregated the prevalence of all outcomes by deafblindness and disability status to measure the relative inequities for children with deafblindness. We also explored these inequities by gender where the sample size permitted.^25^ For all of the outcomes listed above, children with deafblindness were compared with children with other disabilities and children with no disabilities. We modelled the probability of the outcome for different groups with a modified Poisson model,^26^ accounting for the survey design and weighting using the ‘survey’ package in R^27^ and adjusting for age. Indicators are pooled across multiple countries because the sample size of children with deafblindness per country is too small to produce country-specific estimates.

## Results

The descriptive statistics for this study are shown in table 2. Children with deafblindness constituted 0.05% of the sample, and 81.9% of these children reported functional difficulties in at least one other domain. Educational outcomes for children with deafblindness compared with other children with disabilities and children without disabilities are presented in table 3. Only 20% of children aged 3–4 with deafblindness had reached the Early Childhood Development Index (ECDI), which captures children’s development across literacy-numeracy skills, physical development, social-emotional development and learning.^28^ Children with deafblindness were 40% less likely to be developmentally on track using this index compared with children with other disabilities (table 3: adjusted prevalence ratio (aPR) 0.60 (0.38 to 0.95)) and 65% less likely compared with children with no disabilities (aPR 0.35 (0.22 to 0.55)). Boys with deafblindness had lower ECDI scores (11%) than girls without disabilities (30%) and had relatively lower scores compared with other children with disabilities (aPR 0.33 (0.15 to 0.73)) and children without disabilities (aPR 0.20 (0.09 to 0.42)).

**Table 2:** Baseline statistics overall and by group

	Overall	Children without disabilities	Children with other disabilities	Children with deafblindness
Sample size (n (%))	446 233	395 239 (88.5)	50 762 (11.4)	232 (0.05)
Female (n (%))	219 645 (49.2)	196 033 (49.6)	23 491 (46.3)	121 (52.2)
Locality (n (%))				
Rural	267 090 (61.3)	236 058 (61.2)	30 883 (62.2)	149 (65.4)
Urban	167 185 (38.4)	148 453 (38.5)	18 656 (37.5)	76 (33.3)
Other	1605 (0.4)	1450 (0.4)	152 (0.3)	3 (1.3)
Age (in years) (mean (SD))	7.6 (4.7)	7.5 (4.7)	8.9 (4.4)	7.1 (4.8)
Prevalence of additional functional difficulties among children with deafblindness	–	–	–	190 (81.9)

**Table 3:** Education outcomes for children with deafblindness, children with other disabilities and children with no disabilities

	Deafblind (%)	Other disability (%)	aPR (95% CI), other disability*	No disability (%)	aPR (95% CI), no disability*
Early Childhood Development Index: % of those meeting threshold					
All	13/66 (20)	1831/4841 (38)	0.60 (0.38 to 0.95)	75476/109331 (69)	0.35 (0.22 to 0.55)
Girls	9/30 (30)	829/2121 (39)	0.93 (0.56 to 1.54)	38548/54298 (71)	0.53 (0.33 to 0.87)
Boys	4/36 (11)	1002/2720 (37)	0.33 (0.15 to 0.73)	36928/55033 (67)	0.20 (0.09 to 0.42)
Early childhood education attendance					
All	4/57 (7)	779/3983 (20)	0.62 (0.25 to 1.54)	29458/96112 (31)	0.44 (0.17 to 1.19)
Girls	2/25 (8)	331/1726 (19)	0.89 (0.30 to 2.66)	14877/47754 (31)	0.56 (0.18 to 1.68)
Boys	2/32 (6)	448/2257 (20)	0.55 (0.19 to 1.59)	14581/48358 (30)	0.37 (0.12 to 1.16)
Primary school attendance rate					
All	12/59 (20)	13387/20231 (66)	0.30 (0.18 to 0.50)	75888/101157 (75)	0.28 (0.17 to 0.47)
Girls	8/33 (24)	6267/9474 (66)	0.35 (0.21 to 0.58)	38246/50766 (75)	0.33 (0.20 to 0.55)
Boys	4/26 (15)	7120/10757 (66)	0.24 (0.10 to 0.54)	37642/50391 (75)	0.22 (0.09 to 0.50)
Secondary school attendance rate					
All	9/56 (16)	5676/15798 (36)	0.42 (0.20 to 0.87)	46770/96415 (49)	0.35 (0.17 to 0.71)
Girls	5/30 (17)	2707/7384 (37)	0.38 (0.22 to 0.66)	23505/47405 (50)	0.33 (0.19 to 0.56)
Boys	4/26 (15)	2969/8414 (35)	0.45 (0.12 to 1.75)	23265/49010 (47)	0.37 (0.10 to 1.45)

* PR is adjusted by age.

PR, prevalence ratio.

Children with deafblindness had very low attendance in early childhood education programmes (7%) and primary school (20%). Children with deafblindness were less likely to be enrolled in early childhood education compared with children with other disabilities (20%) or children without disabilities (31%), but the difference was not statistically significant. Like ECDI, early childhood education attendance increased proportionally with country income level, which was similar for children with other disabilities and children without disabilities. Additionally, primary school-aged children with deafblindness faced a 2–3 times lower likelihood of enrolment in primary school compared with their counterparts with other disabilities (66% enrolled: aPR 0.30 (0.18 to 0.50)) and those without disabilities (75% enrolled: aPR 0.28 (0.17 to 0.47)). Similarly, only 16% of children with deafblindness were participating in secondary school. Secondary school-aged children with deafblindness were over half as likely to be in school as children with other disabilities (36% enrolled: aPR 0.42 (0.20 to 0.87)) and 65% less likely than children without disabilities (49% enrolled: aPR 0.35 (0.17 to 0.71)). This association was worse for girls with deafblindness compared with girls with other disabilities (aPR 0.38 0.22 to 0.66)) and girls without disabilities (aPR 0.33 (0.19 to 0.56).

Children with deafblindness exhibited poorer health outcomes, particularly when compared with children without disabilities. There was a higher prevalence of diarrhoea in the last 2 weeks among children with deafblindness compared with children without disabilities (table 4: aPR 1.48 (1.09 to 2.00)). Moreover, young children with deafblindness were twice as likely to experience wasting compared with children with other disabilities (aPR 2.08 1.47 to 2.96)) and nearly three times more likely than children without disabilities (aPR 2.79 (1.99 to 3.92)). These inequities were starker for boys, compared with children with other disabilities (stunting: aPR 1.23 (1.01 to 1.50); wasting: aPR 2.77 (1.95 to 3.92)) and without disabilities (stunting: aPR 1.39 1.11 to 1.72); wasting: aPR 3.47 (2.46 to 4.89)). Young children with deafblindness were also more likely to be stunted than children without disabilities (aPR 1.24 (1.03 to 1.50), though this difference was not significant compared with children with other disabilities (aPR 1.07 (0.90 to 1.28). Children with deafblindness were as likely to have health insurance as the other groups. Similarly, there were no significant differences in the prevalence of or care seeking for ARI or diarrhoea between the three groups.

**Table 4:** Health outcomes among children with deafblindness compared with children with other disabilities and no disabilities

	Deafblind (%)	Other disability (%)	aPR (95% CI), other disability*	No disability (%)	aPR (95% CI), no disability*
Health outcomes					
ARI in the past 2 weeks					
All	35/105 (33)	2709/8083 (34)	0.96 (0.76 to 1.22)	39697/157307 (25)	1.20 (0.97 to 1.49)
Girls	16/51 (31)	1167/3594 (32)	0.93 (0.63 to 1.38)	19637/77888 (25)	1.11 (0.75 to 1.64)
Boys	19/54 (35)	1542/4489 (34)	1.00 (0.69 to 1.44)	20060/79419 (25)	1.29 (0.93 to 1.80)
Diarrhoea in the past 2 weeks					
All	23/104 (22)	1437/8043 (18)	1.09 (0.81 to 1.46)	16216/156941 (10)	1.48 (1.09 to 2.00)
Girls	13/50 (26)	624/3569 (17)	1.26 (0.83 to 1.90)	7748/77699 (10)	1.71 (1.13 to 2.59)
Boys	10/54 (19)	813/4474 (18)	0.91 (0.53 to 1.59)	8468/79242 (11)	1.25 (0.72 to 2.18)
Stunted					
All	40/88 (45)	3000/7793 (38)	1.07 (0.90 to 1.28)	40996/154102 (27)	1.24 (1.03 to 1.50)
Girls	18/43 (42)	1355/3471 (39)	0.91 (0.66 to 1.25)	20011/76358 (26)	1.11 (0.83 to 1.49)
Boys	22/45 (49)	1645/4322 (38)	1.23 (1.01 to 1.50)	20985/77744 (27)	1.39 (1.11 to 1.72)
Wasted					
All	19/87 (22)	587/7799 (8)	2.08 (1.47 to 2.96)	7663/153779 (5)	2.79 (1.99 to 3.92)
Girls	6/42 (14)	261/3478 (8)	1.38 (0.71 to 2.68)	3450/76212 (5)	1.99 (1.09 to 3.66)
Boys	13/45 (29)	326/4321 (8)	2.77 (1.95 to 3.92)	4213/77567 (5)	3.47 (2.46 to 4.89)
Access to healthcare					
Health insurance					
All	19/229 (8)	5911/50534 (12)	1.18 (0.84 to 1.65)	44167/387809 (11)	1.10 (0.78 to 1.54)
Girls	11/120 (9)	2572/23382 (11)	1.54 (1.06 to 2.25)	21888/192368 (11)	1.39 (0.94 to 2.06)
Boys	8/109 (7)	3339/27152 (12)	0.88 (0.53 to 1.45)	22279/195441 (11)	0.85 (0.52 to 1.41)
Sought care for ARI in the past 2 weeks					
All	18/34 (53)	1457/2649 (55)	1.12 (0.84 to 1.50)	22062/37146 (59)	1.14 (0.86 to 1.53)
Girls	11/21 (52)	604/1150 (53)	1.11 (0.72 to 1.71)	10787/18291 (59)	1.11 (0.76 to 1.63)
Boys	7/13 (54)	853/1499 (57)	1.24 (0.81 to 1.91)	11275/18855 (60)	1.22 (0.80 to 1.86)
Sought care for diarrhoea in the past 2 weeks					
All	10/23 (43)	815/1437 (57)	0.79 (0.44 to 1.41)	8970/16199 (55)	0.83 (0.47 to 1.49)
Girls	5/13 (38)	336/624 (54)	0.72 (0.32 to 1.58)	4291/7738 (55)	0.76 (0.36 to 1.60)
Boys	5/10 (50)	479/813 (59)	0.90 (0.46 to 1.75)	4679/8461 (55)	0.91 (0.47 to 1.79)

* PR is adjusted by age.

ARI, acute respiratory infection; PR, prevalence ratio.

Well-being outcomes are shown in table 5. In general, there was no discernible difference in the likelihood of poverty among children with deafblindness compared with their counterparts with other disabilities or those without disabilities.

**Table 5:** Well-being outcomes for children with deafblindness, children with other disabilities and children with no disabilities

	Deafblind (%)	Other disability (%)	aPR (95% CI), other disability*	No disability (%)	aPR (95% CI), no disability[Table-fn T5_FN1]
Lives in poverty					
All	115/229 (50)	25188/50534 (50)	0.99 (0.86 to 1.15)	180437/387809 (47)	1.08 (0.94 to 1.25)
Girls	60/120 (50)	11575/23382 (50)	0.98 (0.77 to 1.23)	89195/192368 (46)	1.08 (0.86 to 1.34)
Boys	55/109 (50)	13613/27152 (50)	1.01 (0.88 to 1.16)	91242/195441 (47)	1.09 (0.95 to 1.25)
Inadequate supervision					
All	44/104 (42)	3459/7910 (44)	0.82 (0.70 to 0.96)	45845/158951 (29)	0.92 (0.78 to 1.07)
Girls	22/51 (43)	1586/3501 (45)	0.80 (0.63 to 1.03)	22647/78742 (29)	0.91 (0.72 to 1.15)
Boys	22/53 (42)	1873/4409 (42)	0.83 (0.68 to 1.02)	23198/80209 (29)	0.93 (0.76 to 1.13)
Violent discipline					
All	100/195 (51)	36790/42967 (86)	0.60 (0.51 to 0.71)	274600/340289 (81)	0.62 (0.52 to 0.73)
Girls	46/98 (47)	16758/19735 (85)	0.56 (0.41 to 0.77)	135234/169339 (80)	0.57 (0.41 to 0.78)
Boys	54/97 (56)	20032/23232 (86)	0.65 (0.56 to 0.75)	139366/170950 (82)	0.67 (0.58 to 0.77)
Lives with both parents					
All	151/220 (69)	31285/47692 (66)	0.96 (0.90 to 1.02)	263862/377838 (70)	0.94 (0.89 to 1.00)
Girls	80/115 (70)	14049/22057 (64)	1.00 (0.90 to 1.11)	128679/187400 (69)	0.99 (0.89 to 1.10)
Boys	71/105 (68)	17236/25635 (67)	0.92 (0.84 to 1.00)	135183/190438 (71)	0.90 (0.83 to 0.98)
Child’s birth is registered					
All	51/105 (49)	4919/8062 (61)	0.90 (0.72 to 1.12)	113373/156467 (72)	0.87 (0.71 to 1.07)
Girls	22/51 (43)	2101/3580 (59)	0.87 (0.66 to 1.15)	55740/77446 (72)	0.84 (0.65 to 1.08)
Boys	29/54 (54)	2818/4482 (63)	0.93 (0.74 to 1.18)	57633/79021 (73)	0.90 (0.72 to 1.13)

* PR is adjusted by age.

PR, prevalence ratio.

For children aged 2–14 years, children with deafblindness were less likely to encounter violent discipline from their caregivers, as reported by caregivers, when compared with children with other disabilities and those without disabilities. However, it is important to note that violent discipline was prevalent across all groups, affecting more than 50% of all children.

About half of young children with deafblindness (2–4 years) had their births officially registered, but this difference was not significant.

Notably, there was no distinction between children with deafblindness and those with other or no disabilities in terms of having both biological parents present in their households, though boys with deafblindness were less likely to live with their parents compared with children without disabilities (aPR 0.90 (0.83 to 0.98)).

## Discussion

This is the largest known study examining quantitative indicators related to the health, education and well-being of children with deafblindness from low- and middle-income countries, compiling data from 36 countries. Using internationally comparable data, we show that the situation for children with deafblindness is often worse compared with children with other disabilities and children without disabilities across health and education indicators. Deafblind children were also more likely to be stunted, wasted or have diarrhoeal disease and less likely to attend school and had lower scores on the Early Childhood Development Index compared with children with other disabilities. However, there were no significant differences in health insurance coverage, care seeking for ARI or diarrhoea, living in poverty, living with parents or birth registration for children with deafblindness compared with either group. Compared with both children with other disabilities and children without disabilities, children with deafblindness were less likely to have reported experiencing violent discipline by caregivers. These results show that there are particular areas of disadvantage for children with deafblindness and that further action is needed to improve the health and education outcomes of children with deafblindness.

There is limited literature examining the health of children with deafblindness. Our findings show children with deafblindness have significantly worse malnutrition outcomes, even compared with other children with disabilities—who are known to be at greater risk of stunting and wasting.^29^ Poverty may explain part of this difference: although our results did not show a difference between children with and without deafblindness, it is likely to underestimate poverty among households. Importantly, studies in settings such as Georgia and India have found households with persons with deafblindness experience high disability-related extra costs (eg, for additional healthcare and education supports). ^30 31^There may be other barriers to adequate nutrition, including exclusion from school-based feeding programmes due to lower school attendance.^32^ Moreover, these findings largely echo other studies in the MICS that have shown children with disabilities have greater health needs and prevalence of childhood illnesses.^33 34^ While our data showed that children with deafblindness did not have significant differences in care seeking when ill or health insurance coverage, our small sample size and overall low health insurance coverage (less than 15% coverage for all groups) may have impacted our ability to detect inequities for children with deafblindness. Furthermore, these results merely show access to care and incidence of illness, rather than any indication of the quality of services children with deafblindness receive. Future research should highlight these experiences and trial interventions, as it is important that health systems adequately prepare to support and treat children with deafblindness. For example, ensuring deafblind interpretation in the clinical setting, training health workers about deafblindness and communication and ensuring health information is available in accessible formats can support better health among people with deafblindness.^35^

Moreover, our results showing that children with deafblindness were less likely to have attended education from early childhood to secondary school reflect the literature that shows that children with disabilities face barriers in accessing education. For example, a 2017 study of children with deafblindness in the USA showed that only 30% of children with deafblindness participated in early childhood education.^8^ While this has doubled in the past decade in the USA,^8^ our findings of low attendance rates in middle- and low-income settings emphasise the need to address the barriers to education for children with deafblindness. Since this study only measured attendance, it is also important to consider the quality of education children with deafblindness receive. The UN Convention on the Rights of Persons with Disabilities emphasises the need for inclusive mainstream education, but because of the barriers children with deafblindness experience, they may be segregated into specialised schools or left without appropriate support in mainstream education.^6 35^ Increasing the availability of assistive technology, deafblind interpretation, training teachers and providing greater government support for inclusive education may support greater school attendance and quality of education for children with deafblindness.^35^

The finding that children with deafblindness were less likely to experience violent discipline by caregivers is surprising, since other literature has shown children with disabilities are at increased risk of violence.^36 37^ Similarly, much of the literature surrounding deafblindness discusses the vulnerability of this population to various risks, including violence.^38^ Therefore, our finding that children with deafblindness experience significantly less caregiver violent discipline may be an artefact of several factors. First, violent discipline towards a child with significant disabilities may be particularly prone to underreporting, especially since violent discipline is captured through caregiver response. Second, several actions considered as ‘violent discipline’ may be viewed as culturally appropriate ways of raising children (ie, hitting and spanking). Therefore, lack of violent discipline for these children could be more indicative of ‘giving up’ on the child, rather than providing the usual course of discipline. Finally, this measure of discipline doesn’t include disability-specific forms of discrimination and punishment, such as name-calling, exclusion, neglect and denial of care/support.^39^ Further research should distill the relationship and significance of this finding, although it is important to note that violent discipline was prevalent overall, affecting more than 50% of all children in the study.

### Strengths and limitations

The MICS provide data on nearly 450 000 children from 36 countries on key Sustainable Development Goals indicators, which can help to ensure that children with deafblindness are not ‘left behind’ in development. Still, there are some important limitations to consider. First, the small sample size was underpowered to examine country inequities and instead relied on a pooled result. This may mask any differences between countries—for example, due to different policies and availability of services. Additionally, as described above, some indicators used in MICS may not fully capture the experience of children with deafblindness. As such, inequalities between children with deafblindness and other groups are likely underestimated. Further, although the definition of the variable for deafblindness used in this analysis was agreed upon by the World Federation of Deafblindness and partners as the best strategy given available data, it does not fully align with the agreed definition of deafblindness, nor capture the experience of those with mild deafblindness who may also face barriers to participation. Further action may be needed to develop recommendations and guidelines for measuring deafblindness and outcomes among people with deafblindness in national surveys in the future. Finally, a high proportion of the children with deafblindness also reported functional difficulties in other domains (89%), but the study was underpowered to analyse these children separately from those who exclusively reported difficulties seeing and hearing.

## Conclusion

In conclusion, our study showed children with deafblindness face exclusion on numerous indicators of health, education and well-being and are often worse off compared with children with other disabilities and children without disabilities. Children with deafblindness constitute a particular group within children with disabilities, and greater attention must be paid to ensure that their unique needs are served in efforts to improve health, education and social inclusion for children with disabilities. Without adequate inclusion of children with deafblindness, we will not achieve our global goals of ‘leaving no one behind’.

## Data Availability

Data may be obtained from a third party and are not publicly available.
